# Diffuse large B-cell lymphoma with continuously elevated immunoglobulin M following treatment: a case report with pathologic, immunophenotypic, and molecular analyses

**DOI:** 10.3389/fgene.2023.1228372

**Published:** 2023-11-06

**Authors:** Fei Xiao, Yong-Mei Cai, Jian-Chen Fang, Yan-Ying Shen, Bao-Hua Yu, Yi-Wei Zhang, Di Zhu, Zi-Hua Li, Guo-Qing Li, Jian Hou, Min-Yue Zhang, Hong-Hui Huang

**Affiliations:** ^1^ Department of Hematology, Ren Ji Hospital, School of Medicine, Shanghai Jiao Tong University, Shanghai, China; ^2^ Department of Pathology, Ren Ji Hospital, School of Medicine, Shanghai Jiao Tong University, Shanghai, China; ^3^ Department of Pathology, Fudan University Shanghai Cancer Center, Shanghai, China; ^4^ Shanghai Rightongene Biomedical Technology Co., Ltd., Shanghai, China

**Keywords:** diffuse large B-cell lymphoma, Waldenström macroglobulinemia, immunoglobulin M, immunoglobulin heavy-chain sequencing, *MYD88* mutation

## Abstract

A rare subtype of diffuse large B-cell lymphoma (DLBCL) has been reported to be accompanied by elevated immunoglobulin M (IgM) paraprotein in the serum at diagnosis, called as IgMs-DLBCL. The monoclonal IgM paraprotein disappears soon after treatment in most of these patients. Here, we described a DLBCL patient with continuously elevated IgM following therapy. A 59-year-old male was diagnosed with DLBCL (GCB subtype *per* Hans algorithm, stage IA) with involvement of the right cervical lymph node. After six cycles of immuno-chemotherapy with the R-CHOP regimen, complete metabolic remission was achieved, but an elevated level of serum IgM persisted. To investigate the origin of elevated IgM, pathologic, immunophenotypic, and molecular analyses of lymph node and bone marrow (BM) samples were performed pre- and post-treatment. BM infiltration of lymphoplasmacytic cells, and a typical immunophenotypic profile by flow cytometry supported the diagnosis of Waldenström macroglobulinemia (WM). The MCD subtype of DLBCL was identified by next-generation sequencing of the lymph node at initial diagnosis characterized by co-occurring point mutations in *MYD88* ^*L265P*^ and *CD79B.* Additionally, two different dominant clonotypes of the immunoglobulin heavy chain (*IGH*) were detected in the lymph node and BM by *IGH* sequencing, which was *IGHV* 3–11*06/*IGHJ* 3*02 and *IGHV* 3–11*06/*IGHJ* 6*02, respectively, speculating to be two independent clonal origins. This study will provide a panoramic understanding of the origin or biological characteristics of DLBCL co-occurring with WM.

## Introduction

Diffuse large B-cell lymphoma (DLBCL) is the most common subtype of non-Hodgkin’s lymphoma (NHL), accounting for approximately 31% of adult cases (Swerdlow et al., 2016). By gene expression profiling (GEP), DLBCL can be divided into two main groups with substantially different outcomes: the activated B-cell type (ABC-type) and germinal center B-cell type (GCB-type). Recently, Cox et al. (2022) reported a rare subtype of DLBCL accompanied by elevated immunoglobulin M (IgM) paraprotein in the serum at diagnosis, called as IgMs-DLBCL, which was characterized by the non-germinal center B-cell-like (non-GCB) type, poor prognosis, and disappearance of IgM paraprotein after chemotherapy (Cox et al., 2014; Cox et al., 2022). Mutations in *TP53*, *MYD88*, and *CD79B*, as well as the rearrangement of the immunoglobulin heavy-chain (*IGH*) gene, were found in a small subset of IgMs-DLBCL (Cox et al., 2022).

Waldenström macroglobulinemia (WM) is a rare indolent B-cell neoplasm that was first reported by the Swedish physician Jan G. Waldenström in 1944 and was primarily characterized by bone marrow (BM) infiltration with lymphoplasmacytic cells along with IgM monoclonal gammopathy (WALDENSTRÖM, 1944; Owen et al., 2003). Approximately more than 90% WM patients have the *MYD88* ^L265P^ mutation (Treon et al., 2012; Varettoni et al., 2013). It has been reported that DLBCL occurs in 2%–10% WM patients at diagnosis or several years after diagnosis, and the disease can either be *de novo* or result from histological transformation (HT) of pre-existing WM clone (Lin et al., 2003; Leleu et al., 2009).

Here, we reported a patient with DLBCL co-occurring with WM who harbored unique clinical features and genetic alterations. They were quite distinct from those reported by Cox MC et al. By the genetic analysis, a WM clone origin independent of DLBCL was detected, which might account for a continuously high level of serum IgM after immuno-chemotherapy.

## Case presentation

A 59-year-old male complaining of painless enlargement of the right cervical lymph node for several months was admitted to our institution in February 2022. The patient had no symptoms of fever, night swear, weight loss, dizziness, headache, numbness or weakness in the limbs, Raynaud’s phenomenon, vision deterioration, or bulky adenopathy. Fluorine-18-fluorodeoxyglucose (FDG) positron emission tomography–computed tomography (PET/CT) scan showed enlargement of the right cervical lymph node (53 mm × 34 mm) with increased metabolic activity (SUV_max_ 27.7) (Supplementary Figures S1A–C). A right cervical lymph node biopsy was carried out. Pathological findings suggested DLBCL. Immunohistochemistry (IHC) staining demonstrated that the tumor cells were positive for CD20, CD10, IgM, and BCL-6, and partially positive for MUM-1, c-MYC (30%), and P53 (30%–40%) (Supplementary Figures S1D–K), while negative for CD3, BCL-2, MNDA, CD5, cyclinD1, CD21, CD23, and AE1/AE3. The proliferation index of Ki-67 was approximately 90%. Epstein–Barr virus (EBV)-encoded small RNA (EBER) was negative. A bone marrow (BM) smear revealed normal hyperplasia with 25.5% mature lymphocytes, and plasmacytoid change was occasionally seen. BM biopsy showed normocellular marrow with no signs of large B-lymphoma cell infiltration. The serological examination demonstrated an elevated serum monoclonal IgM of 18.2 g/L and surge in the value of lactate dehydrogenase (LDH, 537 IU/L), whereas a normal level of beta 2-microglobulin. The blood routine test showed mild anemia [hemoglobin level (Hb) 125 g/L]. The patient was diagnosed with DLBCL, not otherwise specified (GCB type *per* Hans algorithm, stage IA). Thus, (R)-CHOP (rituximab, cyclophosphamide, doxorubicin, vindesine, and prednisone) immune-chemotherapy was started. Due to being positive for HBV-DNA, rituximab was omitted in the first and second cycles until HBV-DNA declines below 20 IU/ml. The patient achieved complete metabolic remission (CMR) evaluated by PET/CT after six cycles of chemotherapy. The patient ceased chemotherapy and received follow-up visit every 3 months. He remained complete remission for DLBCL, and the duration of remission (DOR) was 13 months until manuscript submission. However, during the treatment and follow-up visit, serum IgM persisted elevated (Supplementary Figure S1L), while the levels of LDH and Hb were normal.

**FIGURE 1:**
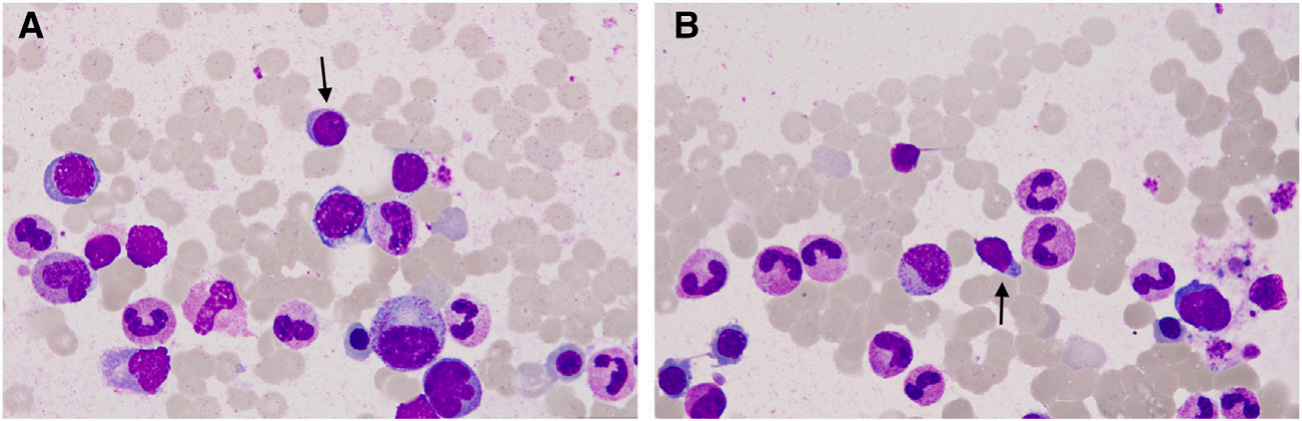
Representative bone marrow (BM) smear images after chemotherapy. **(A, B)** Plasmacytoid change of lymphocytes was observed occasionally in the BM smear (arrow).

**FIGURE 2:**
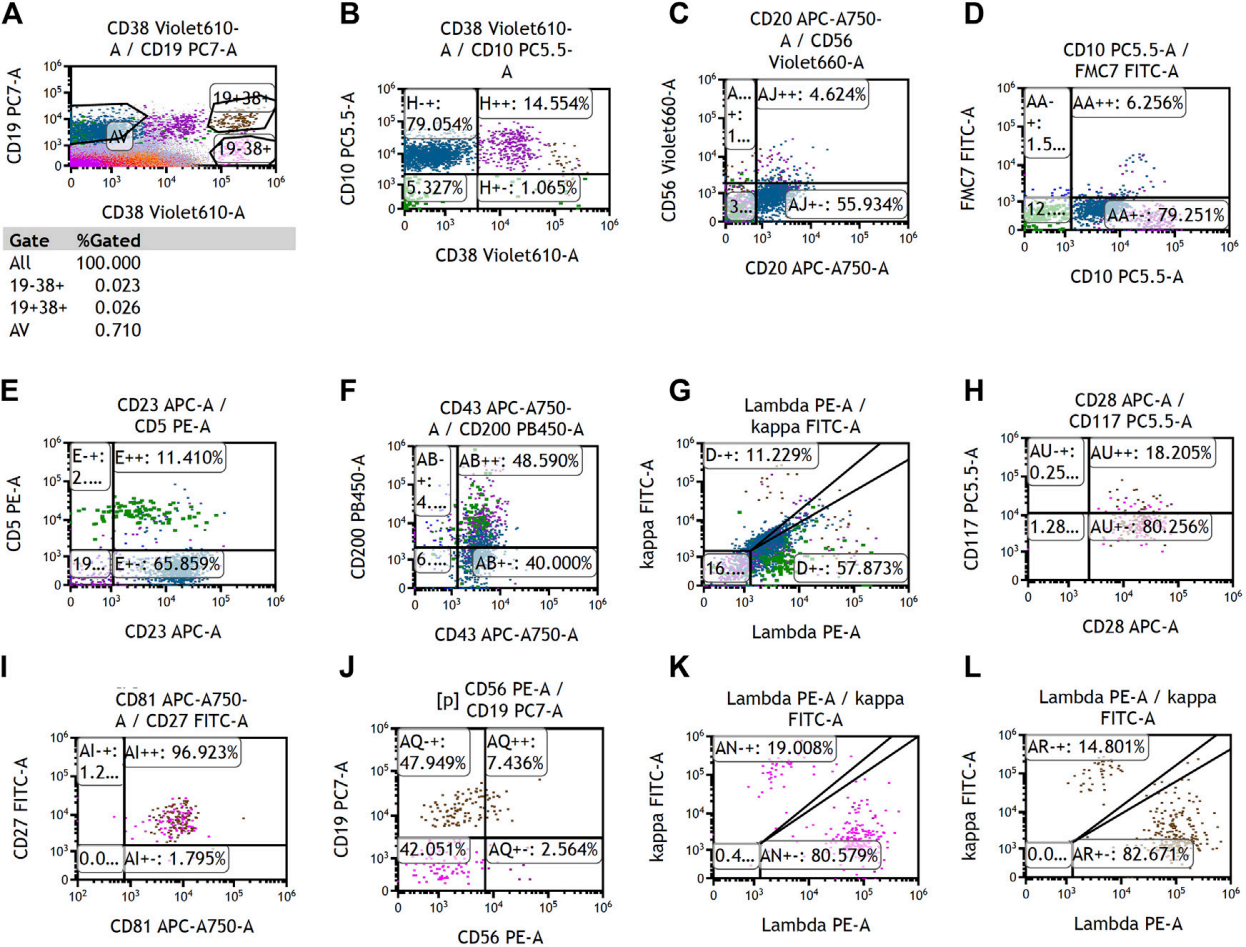
Representative images of multiparameter flow cytometry analysis of the bone marrow after chemotherapy. **(A–G)** Two distinct subgroups of abnormal B-cell populations, which were positive for CD19. **(A, H–L)** One distinct subgroup of abnormal plasma cells, which was positive for CD38.

**FIGURE 3:**
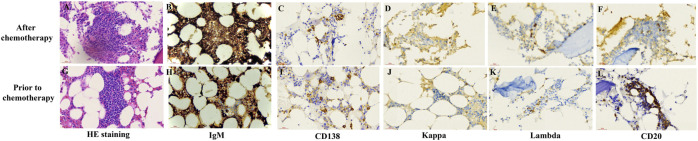
Representative pathological staining images of bone marrow (BM) biopsy after chemotherapy and at initial diagnosis. H&E staining (400X) of the BM biopsy sample after chemotherapy **(A)** and prior to chemotherapy **(G)** showed the infiltration of lymphocytes into bone trabeculae. Immunohistochemistry staining (400X) of the BM biopsy sample after chemotherapy **(B–E)** and prior to treatment **(H–L)**.

To determine the cause of elevated IgM, BM aspiration and biopsy were performed after six cycles of treatment. The plasmacytoid change of lymphocytes was observed occasionally in the BM smear (Figures 1A, B). Furthermore, three distinct subgroups of abnormal cell populations were observed by flow cytometry. One subgroup accounted for approximately 0.24% of nuclear cells with a high side scatter (blue dots circled in Figures 2A–G), which was positive for CD19 (Figure 2A), CD10 (Figures 2B, D), CD20 (Figure 2C), CD23bri (Figure 2E), CD200part (Figure 2F), and CD43 (Figure 2F), and negative for CD22, CD38 (Figures 2A, B), FMC7 (Figure 2D), and CD5 (Figure 2E), without the expression of the cytoplasm immunoglobulin light chain (Figure 2G). Another subgroup accounted for 0.02% of nuclear cells with a low side scatter (green dots circled in Figures 2A–G), and positively expressed CD19 (Figure 2A), CD22dim, CD5 (Figure 2E), CD23 (Figure 2E), CD43 (Figure 2F), and CD200 (Figure 2F), with no expression of CD38 (Figures 2A, B), CD20 (Figure 2C), CD10 (Figures 2B, D), CD11c, and FMC7 (Figure 2D), with lambda light chain being restricted (Figure 2G). The third subgroup was an abnormal plasma cell population, which accounted for approximately 0.06% of nuclear cells (brown and purple dots circled in Figure 2A), positively expressed CD45, CD38 (Figure 2A), CD138, CD28 (Figure 2H), CD27 (Figure 2I), CD81 (Figure 2I), and CD19part (Figure 2J), and negatively expressed CD117 (Figure 2H) and CD56 (Figure 2J), with lambda light chain being restricted (Figures 2K–L). On the other hand, infiltration of lymphocytes into the bone trabeculae was observed in BM biopsy (Figure 3A), and immunohistochemical (IHC) staining showed that the plasmacytoid cells accounted for approximately 5% of nuclear cells and were lambda-restricted. In detail, those cells were positive for IgM (Figure 3B), CD138 (Figure 3C), kappa (Figure 3D), lambda (Figure 3E), Ki-67, and CD10, and partially positive for CD19, CD3, CD34, CD117, TDT, BCL2, and BCL6, while negative for CD20 (Figure 3F), MUM-1, and c-MYC. Furthermore, the BM biopsy sample prior to treatment was retrospectively examined again. Lymphocytes also infiltrated into the bone trabeculae (Figure 3G). The plasmacytoid cells accounted for approximately 5% of nuclear cells, which were positive for IgM (Figure 3H), CD138 (Figure 3I), kappa (Figure 3J), lambda (Figure 3K), CD20 (Figure 3L), PAX-5, and CD38, and negative for cyclinD1, CD56, CD117, CD3, and CD19 by IHC staining. However, the ratio of kappa: lambda was 1:3, which were highly suspected to be clonality. Thus, after initial diagnosis of DLBCL, another synchronous clone of WM was speculating, evidenced by persisted IgM monoclonal gammopathy and typical bone marrow cytology and the immunophenotype profile.

To further understand the characteristics of this patient, we also performed next-generation sequencing (NGS) and droplet digital polymerase chain reaction (ddPCR) to analyze the mutation profile and *IGH* sequences of the lymph node and BM samples. The detailed methods of NGS and ddPCR were showed in Supplementary Methods S1. Targeted sequencing of 114 DLBCL-related genes is listed in Supplementary Table S1. By targeted sequencing of the lymph node at initial diagnosis, mutations in *CD79B*, *EZH2*, *MYD88*, *BTG2*, *DUSP2*, *ITPKB*, *KRAS*, *LYN*, *SGK1*, *TBL1XR1*, *TCF3*, *B2M*, *CIITA*, *HIST1H1E*, *IGLL5*, and *PCLO* were detected (Supplementary Figure S2) and the MCD subtype of DLBCL was identified characterized by co-occurring point mutations in *MYD88* ^*L265P*^ and *CD79B* (Wright et al., 2020). The variant allele frequency (VAF) of *MYD88* ^*L265P*^ was 51.6% (Table 1). *MYD88* ^*L265P*^ mutation was also detected in the BM sample at initial diagnosis by using ddPCR with a VAF value of 1.8% (Table 1), which was not found in the BM sample by targeted sequencing after chemotherapy. On the other hand, the dominant clone of *IGH* sequences with a ratio of 64.4% in the lymph node sample at initial diagnosis was *IGHV* 3–11*06/*IGHJ* 3*02 by sequencing of *IGH* (Table 1; Supplementary Table S2). The same *IGH* sequence was present in the BM sample prior and following treatment with extremely low ratios of 0.27% and 0.67%, respectively (Table 1), suggesting minimal marrow involvement by DLBCL at initial diagnosis. Moreover, 4 months after completion of chemotherapy, *IGHV* 3–11*06/*IGHJ* 3*02 was not detected (Table 1). Interestingly, another different *IGH* clone, *IGHV* 3–11*06/*IGHJ* 6*02, was also detected both in the BM sample and lymph node samples before treatment with low ratios of 0.15% and 1.2%, respectively. However, this *IGH* sequence became the dominant clone in the BM sample, following chemotherapy with a ratio of 33.94%, which was still the highest clone in the BM sample with a ratio of 17.66% 4 months after completion of chemotherapy. These results indicated that there were two different *IGH* clones both in the BM and lymph node sample. One of the clones (*IGHV* 3–11*06/*IGHJ* 3*02) might be related to DLBCL whereas another (*IGHV* 3–11*06/*IGHJ* 6*02) might be related to WM (Supplementary Figure S3), which probably attributed to the persisted elevation of the serum IgM level post-treatment.

**TABLE 1:** Ratios of *MYD88* ^*L265P*^ and *IGH* clones in this patient.

Sample	Lymph node at initial diagnosis (%)	BM samples at initial diagnosis (%)	BM samples following treatment (%)	BM samples after completion of chemotherapy (%)
*MYD88* ^*L265P*^ mutation	51.6	1.8	0	Not available
*IGHV* 3–11*06/IGHJ 3*02 (DLBCL clone)	64.4	0.27	0.67	0
*IGHV* 3–11*06/*IGHJ* 6*02 (WM clone)	1.2	0.15	33.94	17.66

Abbreviations BM, bone marrow; DLBCL, diffuse large B-cell lymphoma; WM, Waldenström macroglobulinemia.

## Discussion

In this study, we reported a Chinese patient with DLBCL and co-occurring WM, which might originate from two different clones. A series of interesting observations were obtained from this case.

First, IgMs-DLBCL is a rare subtype of DLBCL, which is defined as DLBCL accompanied by the detection of IgM paraprotein in the serum and the cytoplasmic expression of the same subtype of heavy and light chains by Cox et al. (2022). Recently, Cox et al. (2022) retrospectively analyzed 102 IgMs-DLBCL patients and described their clinical characteristics, including prevalence of the non-GCB subtype; recurrent mutations of *TP53, MYD88,* and *CD79B* genes; BCL2 overexpression; and frequent involvement of the central nervous system. Patients with IgMs-DLBCL had inferior prognosis compared with those without paraprotein or with other types of paraprotein. In our case, *MYD88,* and *CD79B* gene mutations were detected, which was consistent with Cox’s cohort. Nevertheless, the COO subtype of DLBCL was GCB type, and overexpression of BCL-2 was not found in lymph node samples by IHC, which were contrary to Cox MC’s study. ABC-type DLBCL usually expressed genes that are characteristic of plasma cells including *IGH* (Wright et al., 2003), suggesting a possible cause of secreted IgM. The secretory mechanism of IgM in GCB-type DLBCL needs further studies.

Second, the serum IgM of our case persisted elevation before and after treatment, whereas Cox et al. (2014) reported that the serum IgM disappeared quickly after R-CHOP treatment in most IgMs-DLBCL patients. For patients with persisted high serum IgM, the author found that IHC of the IgM monoclonal component in DLBCL samples was negative, which meant that secretion of IgM was not related to the DLBCL clone. In our case, the patient achieved CMR after treatment, but the elevated IgM concentration persisted. Thus, we further explored the cause of persistent elevation of serum IgM. The plasmacytoid change of lymphocytes in the BM smear and the detection of small B lymphocytes and abnormal plasma cells in the BM sample by flow cytometry supported the diagnosis of WM in addition to DLBCL. Additionally, we also found that IgM was positive for large lymphocytes in the lymph node and for small lymphocytes in BM biopsy samples by IHC. Collectively, we speculated that IgM might be produced by both DLBCL and WM at initial diagnosis, while a WM clone caused elevation of IgM after completion of chemotherapy.

Third, in several small series and case reports, it had been previously described that patients with DLBCL can occur as a result of histological transformation from several years after WM diagnosis (Lin et al., 2003; Leleu et al., 2009). However, it is extremely rare for these two subtypes of lymphoma to occur simultaneously. To further comprehensively understand this rare phenomenon, DLBCL cases co-occurring with WM (the interval between DLBCL and WM diagnosis less than 6 months) were retrospectively extracted from the Surveillance, Epidemiology, and End Results (SEER) database from 2000 to 2018. Totally, 29 cases with female slight predominance (55.2%) were included for analysis. The median age at initial diagnosis was 69 years old (range 50–89 years old). Most cases (72.4%) were nodal DLBCL. The median overall survival (OS) was 46 months (95% confidence interval, 12.1–79.9 months). There was no significant difference of OS between the patients with DLBCL co-occurring with WM and *de novo* DLBCL patients from the SEER database (Supplementary Figure S4). On the other hand, whether DLBCL co-occurring with WM develops by clonal evolution or as a secondary neoplasm is still controversial. To clarify this issue, abundant studies were conducted by analyzing complementarity determining region 3 (CDR3) in the *IGH* gene based on PCR molecular methods, but the results were discordant (Nakamura et al., 2000; Shimizu et al., 2003; Tojo et al., 2004; Shiseki et al., 2011; Talaulikar et al., 2019), which suggested that in the cases of WM, DLBCL can occur via two mechanisms: clonal evolution and a second malignancy. However, it is not clear which of the two mechanisms is dominant in the pathogenesis of HT of WM to DLBCL. In our case, *IGH* sequencing was applied to determine the clonality of DLBCL and WM. Two different clonotypes of *IGH* were detected in lymph node and BM samples at initial diagnosis by *IGH* sequencing, which were IGHV 3–11*06/IGHJ 3*02 and IGHV 3–11*06/IGHJ 6*02. One of the clones (*IGHV* 3–11*06/*IGHJ* 3*02) might be related to DLBCL whereas another (*IGHV* 3–11*06/*IGHJ* 6*02) might be related to WM. Hence, we speculated that DLBCL and WM co-occurred at initial diagnosis. However, tumor cells of DLBCL were not clonally identical to those of WM, which was consistent with Talaulikar et al. (2019), Shimizu et al. (2003), and Tojo et al. (2004). Our study indicated that not all IgM-DLBCL or DLBCL with *MYD88* ^*L265P*^ mutation a single clonal population. Whether DLBCL harbored a hidden WM clone should be screened in certain cases.

Fourth*, MYD88* encodes a cytosolic adapter protein that plays an essential role in the interleukin-1 and Toll-like receptor signaling pathways, which regulate the activation of numerous pro-inflammatory genes (Yu et al., 2018). *MYD88* ^*L265P*^ mutation is detected in more than 90% WM patients and approximately 15% DLBCL patients (Treon et al., 2012; Varettoni et al., 2013; Schmitz et al., 2018; Lacy et al., 2020). In our case, the *MYD88* ^*L265P*^ mutation was only detected before treatment with VAF of 51.6% in the lymph node and 1.8% in BM. It is difficult to determine whether the mutation originated from the DLBCL clone or WM clone. However, after six cycles of (R)-CHOP treatment, the mutation was not detected in BM, which is possibly due to the limitation in sensitivity of NGS technology. Interestingly, the *MYD88* ^*L265P*^ mutation was detected in this DLBCL case with the GCB subtype, which was contrary to the previous reports that *MYD88* or *CD79B* mutation was often present in the ABC subtype of DLBCL. However, the mechanism was unknown. Recently, Qin et al. (2023) investigated the clinical and molecular differences between DLBCL patients with the MYD88 mutation. It was observed that 17% of the GCB subtype of DLBCL patients had *MYD88* ^*L265P*^ mutation. The *MYD88* ^*L265P*^ mutation can contribute to constitutive NF-κB activation. Classic and alterative NF-κB pathways were also found to be activated in the GCB subtype of DLBCL patients (Compagno et al., 2009). In addition, mutations of BCR/PI3K signaling intermediates (RHOA, GNA13, and SGK1) and NF-kB modifiers (CARD11, NFKBIE, and NFKBIA) were also reported to be enriched in the GCB subtype of DLBCL (Chapuy et al., 2018). However, the mechanism of the *MYD88* alteration involved in the pathogenesis of the GCB subtype of DLBCL remains to be investigated in future study.

Last but not the least, numerous studies had identified genetic subtypes of DLBCL based on shared genomic abnormalities recently, and DLBCL with *MYD88*/*CD79B* alterations were always classified to one cluster, including the MCD/C5/MYD88 cluster (Chapuy et al., 2018; Schmitz et al., 2018; Lacy et al., 2020). *MYD88* and *CD79B* co-mutations were detected in our case at the initial diagnosis of DLBCL, along with mutations of *EZH2*, *BTG2*, *DUSP2*, *ITPKB*, *LYN*, *SGK1*, *TBL1XR1*, *TCF3*, *B2M*, *CIITA*, *HIST1H1E*, and *PCLO*, which were also detected in MCD/C5/MYD88 groups of Chapuy et al. (2018), Schmitz et al. (2018), Lacy et al. (2020), respectively. In addition, mutations of *MYD88* and *EZH2* were detected in our case at the initial diagnosis of DLBCL, which were also significantly enriched in the Depleted category, a subtype of DLBCL based on transcriptomic analysis of the microenvironment of 4,655 DLBCLs in Kotlov et al. (2021)’s study. Compared with other subtypes, patients in the Depleted category had the worst prognosis with a five-year overall survival of approximately 60%. Moreover, Zhao S, et al. proposed a novel histo-molecular classification system (four categories) based on the correlation with morphology of tumor cells and *MYD88* ^*L265P*^ or *CD79B* mutations in primary adrenal DLBCL. These four categories included the neuroendocrine carcinoma (NEC)-like type with *CD79B* mutation, Reed–Sternberg (RS)-like cell type with *MYD88* ^*L265P*^ mutation, biphasic type coexisting mutations of MYD88^L265P^ and *CD79B*, and the common cell type without mutation. The common type showed significantly better survival than other three subtypes (Chen et al., 2020). In the current case, the patient harbored *MYD88* ^*L265P*^ and *CD79B* co-mutations by NGS and displayed compounded morphologic features of NEC-like and RS-like cell types, which was classified the biphasic cell type pattern and might have an inferior prognosis. Therefore, a long-term follow-up was needed for our patient.

In the current study, the patient was diagnosed as coexistence of WM and DLBCL, supported by pathologic, immunophenotypic, and molecular analyses of lymph node biopsy and BM samples. More importantly, to explore the clonality of DLBCL and WM, we conducted *IGH* repertoire analysis with the NGS method, which has higher sensitivity than the conventional PCR method applied in previous studies (Shimizu et al., 2003; Tojo et al., 2004). One of the most important things that our study can note for these genomic classification studies is that tumors are almost never a single clonal population. However, a weak point of our work was the limited case number for IGH and mutational analysis. In addition, the pathogenesis of this rare phenomenon has not been comprehensively elucidated. Whether *MYD88* ^*L265P*^ mutation originated from DLBCL or WM could not be determined in the current study. We will clarify the molecular pathogenesis with more sample sizes or with DLBCL cell lines U2932 expressing CD20 and CD38 by single-cell RNA-sequencing in the future.

Collectively, we reported a Chinese DLBCL patient with persistent elevation of serum IgM. Our finding suggested that WM, another clone independent of DLBCL, might be a possible explanation of elevation of serum IgM. The mechanism underlying the persisted increased level of IgM in DLBCL patients after treatment deserves further investigations in the future, which may provide a panoramic understanding of the origin or biological characteristics of the disease.

## Data Availability

The data cannot be publicly released due to concerns regarding patient confidentiality. Requests to access these datasets should be directed to the corresponding authors.

## Supplementary material

SUPPLEMENTARY METHODS S1 Detailed methods of next-generation sequencing and droplet digital polymerase chain reaction.

SUPPLEMENTARY TABLE S1 List of diffuse large B-cell lymphoma gene panel in next gene sequencing (NGS) and the results of NGS for lymph node and bone marrow samples.

SUPPLEMENTARY TABLE S2 Results of IgH sequencing of lymph node and bone marrow samples.

SUPPLEMENTARY FIGURE S1 The positron emission tomography-computed tomography (PET/CT) images, pathological analysis of lymph node at initial diagnosis and serum IgM level change. **(A–C)** The PET/CT images of patient at baseline; **(D–K)** Images of H&E staining (400X) **(D,E)** and immunohistochemistry staining (400X) **(F–K)** of lymph node at initial diagnosis. Arrows indicated R-S like tumor cells. **(L)** Serum IgM level change at initial diagnosis, during chemotherapy and follow-up visit.

SUPPLEMENTARY FIGURE S2 Mutational landscape among the lymph node at diagnosis and the BM pre–treatment.

SUPPLEMENTARY FIGURE S3 Graphical abstract of development of two different clones, including diffuse large B-cell lymphoma and Waldenström macroglobulinaemia.

SUPPLEMENTARY FIGURE S4 Comparison of overall survival (OS) between patients with de novo diffuse large B-cell lymphoma (DLBCL) and DLBCL co–occurring with WM from the Surveillance, Epidemiology, and End Results (SEER) database.
