# Supplementary figures and images for: Diffuse large B-cell lymphoma with continuously elevated immunoglobulin M following treatment: a case report with pathologic, immunophenotypic, and molecular analyses
Source: Front Genet. 2023 Nov 6;14:1228372. doi: 10.3389/fgene.2023.1228372 (PMC10657880; doi:10.3389/fgene.2023.1228372)

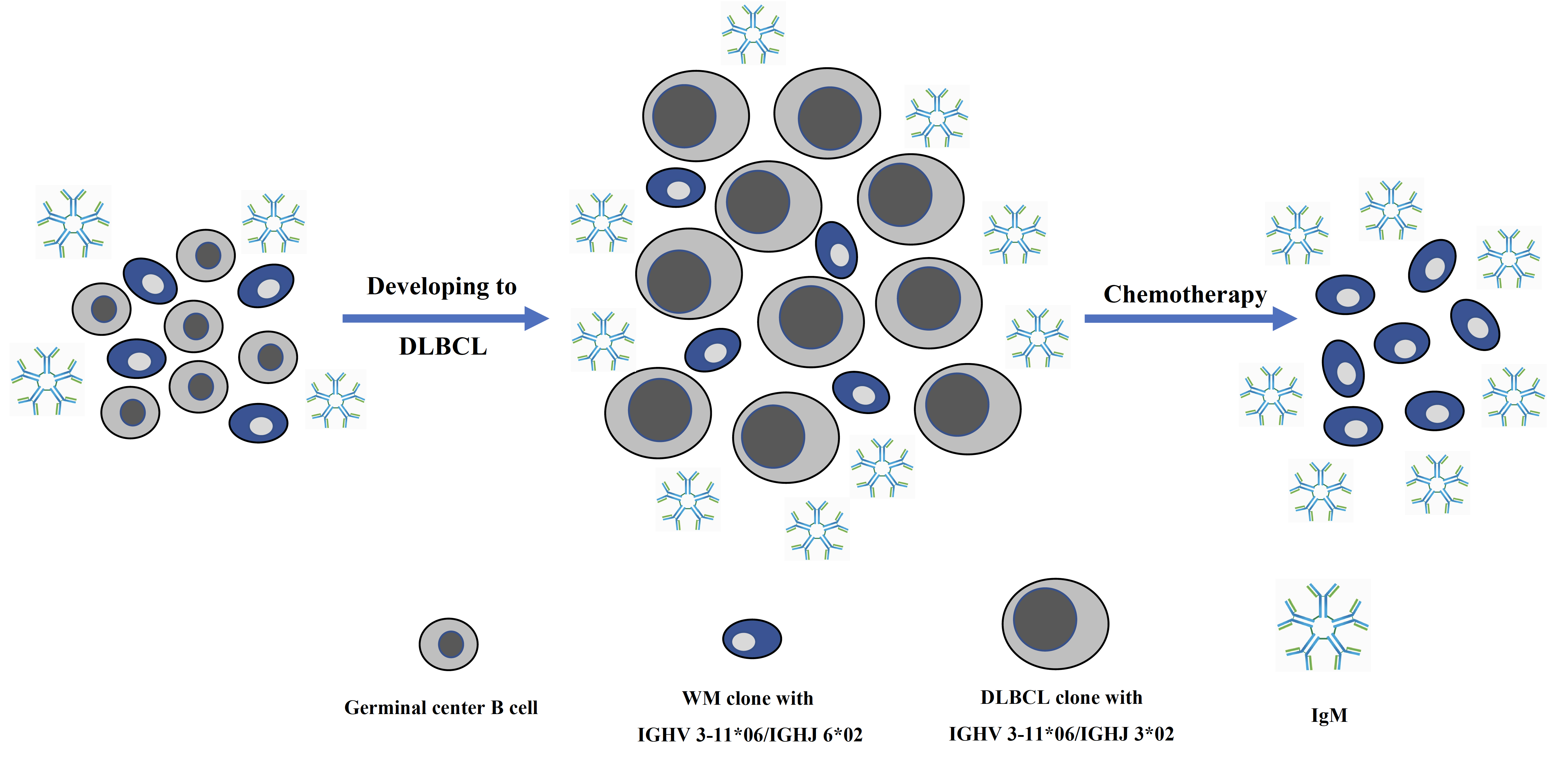

Supplement: Supplementary file 1 [file Image3.JPEG]

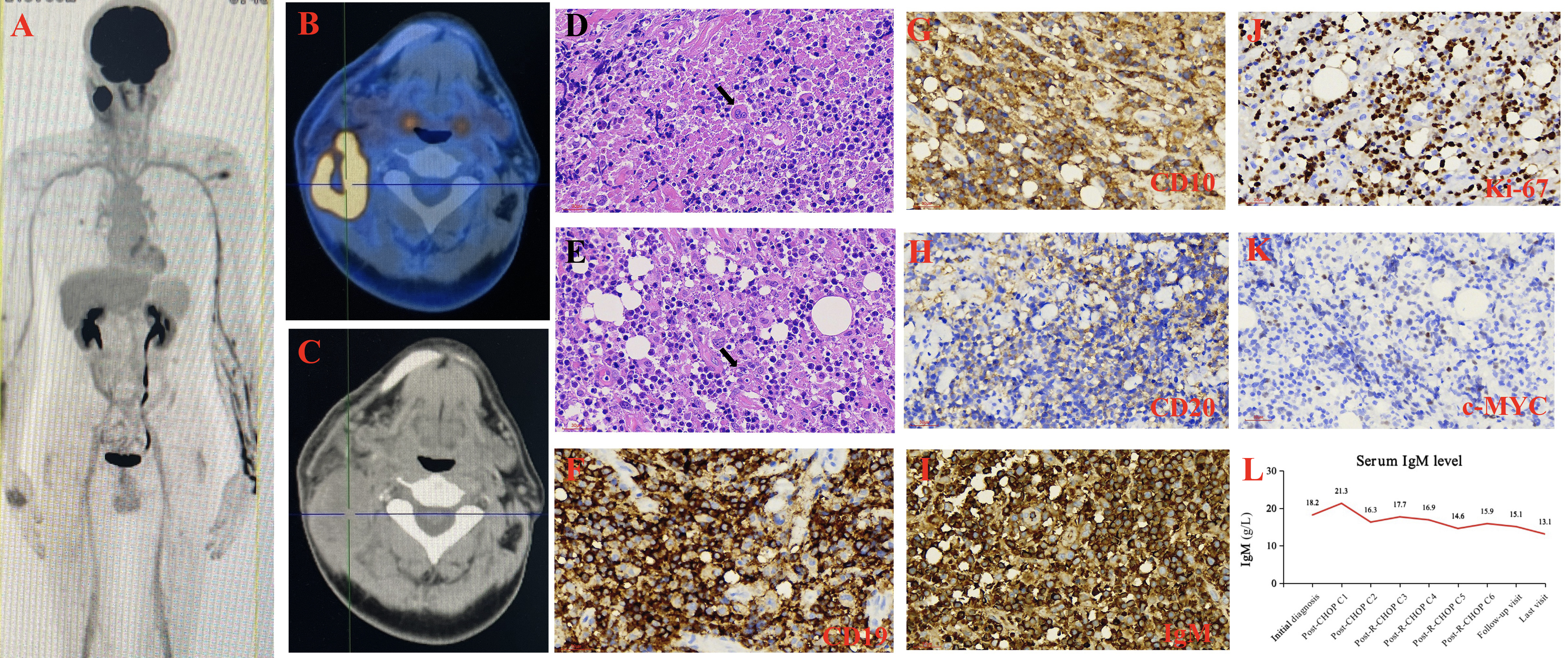

Supplement: Supplementary file 4 [file Image1.JPEG]

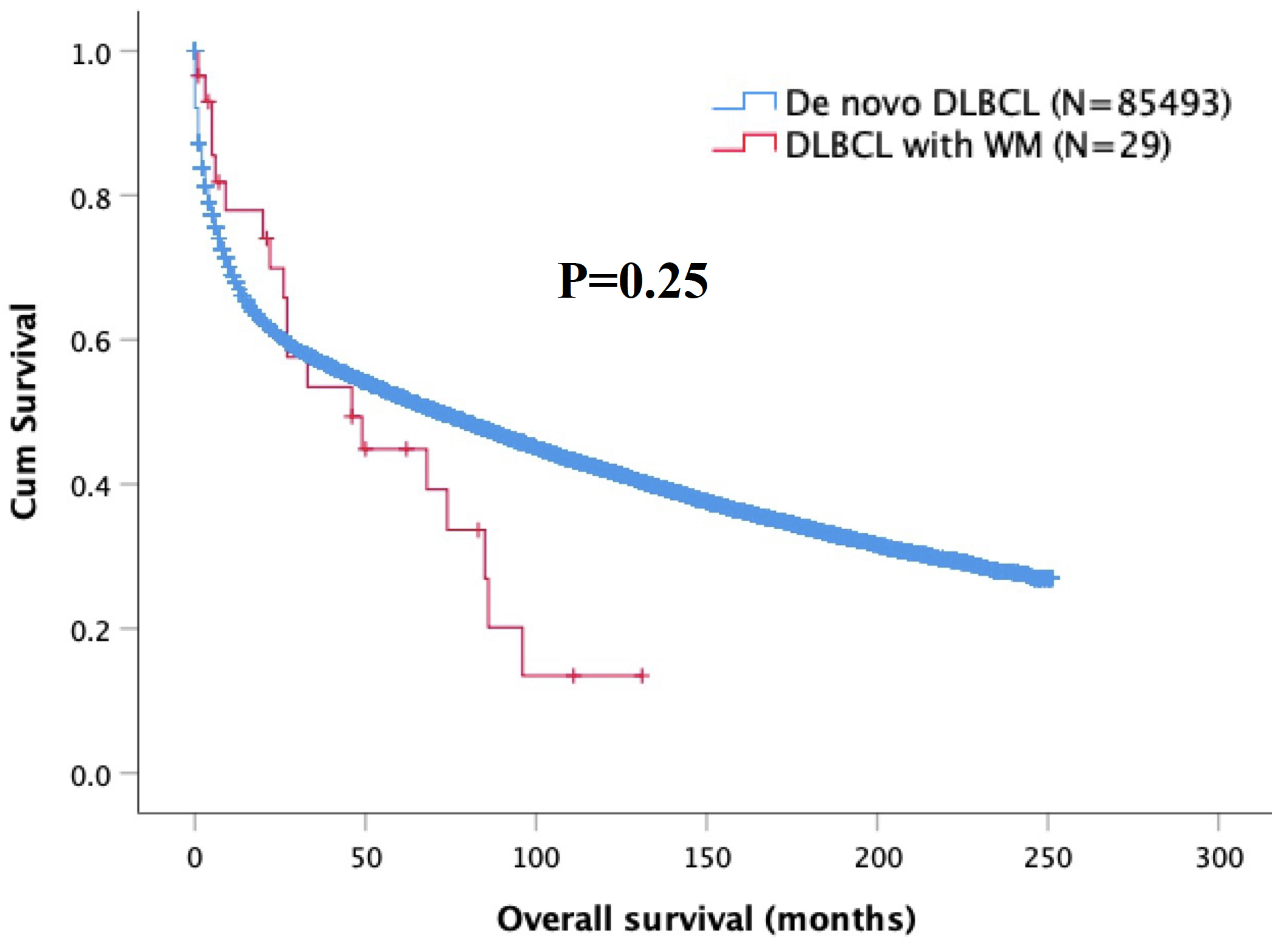

Supplement: Supplementary file 5 [file Image4.JPEG]

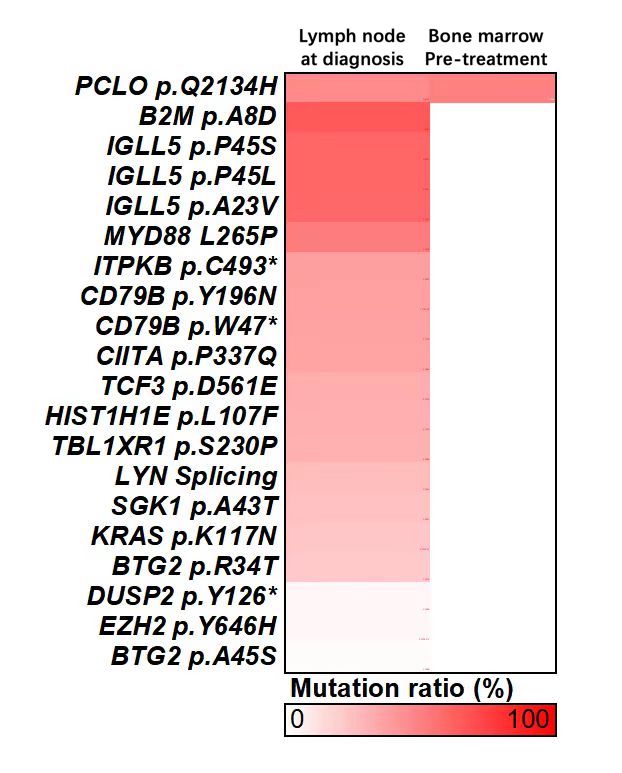

Supplement: Supplementary file 6 [file Image2.JPEG]
