# Supplementary material for: Diffuse large B-cell lymphoma with continuously elevated immunoglobulin M following treatment: a case report with pathologic, immunophenotypic, and molecular analyses
Source: Front Genet. 2023 Nov 6;14:1228372. doi: 10.3389/fgene.2023.1228372 (PMC10657880; doi:10.3389/fgene.2023.1228372)
Supplement: Supplementary file 7 [file DataSheet1.pdf]

Supplemental Methods

Targeted sequencing of 114 DLBCL-related genes (Supplementary table 1) were carried out on Nova-seq (Illumina, San Diego, CA, USA). Mutations were filtered by Shanghai Rightongene Biotechnology Co., Ltd. (Shanghai, China). Mutations meeting the following conditions were screened out: (1) minor allele frequencies ≥ 0.001 according to the 1000 Genomes Project (1) and ExAC (2) databases; (2) variant allele frequency (VAF) ≥ 1%; (3) mutations not in coding regions or splicing sites; (4) synonymous mutations. The sequencing of *IGH* sequence was performed on MiSeq (Illumina, San Diego, CA, USA) platform. Sequences were spliced, removed of redundancy and counted by VSEARCH (3). Then, the sequences were aligned by IGBLASTN (4). Finally, fragments with more than 98% similarity were considered to be the same clone, and corresponding ratio was calculated based on the proportion in the total number of sequences. Droplet digital PCR was performed on QX200 droplet digital PCR system according to the manufacturer's instructions (Bio-Rad, CA, USA).

Reference

1. Abecasis GR, Altshuler D, Auton A, Brooks LD, Durbin RM, Gibbs RA, et al. A map of human genome variation from population-scale sequencing. Nature. 2010;467(7319):1061-73.

2. Lek M, Karczewski KJ, Minikel EV, Samocha KE, Banks E, Fennell T, et al. Analysis of protein-coding genetic variation in 60,706 humans. Nature. 2016;536(7616):285-91.

3. Rognes T, Flouri T, Nichols B, Quince C, Mahe F. VSEARCH: a versatile open source tool for metagenomics. PeerJ. 2016;4:e2584.

4. Ye J, Ma N, Madden TL, Ostell JM. IgBLAST: an immunoglobulin variable domain sequence analysis tool. Nucleic Acids Res. 2013;41(Web Server issue):W34-40.
